# An fMRI study of decision-making under conflict in individuals with autism spectrum condition

**DOI:** 10.1192/j.eurpsy.2021.1954

**Published:** 2021-08-13

**Authors:** S. Tei, J. Fujino

**Affiliations:** 1 Department Of Psychiatry, Kyoto University, Kyoto, Japan; 2 Institute Of Applied Brain Sciences, Waseda University, Saitama, Japan; 3 School Of Human And Social Sciences, Tokyo International University, Saitama, Japan; 4 Medical Institute Of Developmental Disabilities Research, Showa University, Tokyo, Japan; 5 Department Of Psychiatry And Behavioral Sciences, Tokyo Medical and Dental University, Tokyo, Japan

**Keywords:** flexibility, decision making, autism, fMRI

## Abstract

**Introduction:**

Individuals with autism spectrum condition (ASC) frequently report difficulties in social communications, combined with restricted inflexible behaviors. However, it is unclear whether this rigidity is pervasive across cognitive flexibility (CF) and affective flexibility (AF) in situations which resolve different social conflicts.

**Objectives:**

To study CF and AF levels and associated brain activity in individuals with ASC.

**Methods:**

Individuals with ASC and with typical development (TD) performed a moral dilemma task during functional magnetic resonance imaging. For CF, participants made decisions on (1) whether to enforce result-oriented actions to prioritize social/public benefits; and (2) judged whether these actions are right or wrong. For AF, participants made decisions on (1) whether to permit social norm/rule violations in sympathy-evoking situations; and (2) permit these identical violations in no sympathy-evoking situations. We calculated participants’ CF/AF levels by computing the switching-rate of decisions in CF/AF sessions (switching was defined as: CF, judging the actions as wrong but choosing to enforce the action in the same vignette; AF, judging the violations as not permissible in a no sympathy-evoking circumstance, but permissible in a sympathy-evoking circumstance).

**Results:**

For CF, ASC participants showed a marked decrease in CF switching-rates compared to TD participants (p < 0.05), and in corresponding brain activity for executive functioning. For AF, although the AF switching rate difference was non-significant, we observed unique brain activities in each group (e.g., TD activation of the greater dorsomedial-prefrontal cortex and ASC activation of the cingulate cortex).

**Conclusions:**

Our results suggest ASC inflexibility may be further characterized by both CF and AF.

**Disclosure:**

No significant relationships.

